# Pediatric Graves’ disease: insights into clinical characteristics and treatment outcomes

**DOI:** 10.20945/2359-4292-2025-0017

**Published:** 2025-08-04

**Authors:** Akshatha Anand, Vani Hebbal Nagarajappa, Raghupathy Palany

**Affiliations:** 1 Division of Pediatric and Adolescent Endocrinology, Indira Gandhi Institute of Child Health, Bengaluru, India

**Keywords:** Graves’ disease, Hyperthyroidism, Antithyroid agents

## Abstract

**Objective:**

To identify early manifestations of Graves’ disease in young patients and its treatment outcomes.

**Subjects and methods:**

This was a hospital-based review of case records of 47 children (aged 1 month to 18 years) with Graves’ disease from 2011 to 2022. Data were summarized and statistically analyzed.

**Results:**

This study included 47 patients with Graves’ disease, of whom 31 (66%) were girls. The average age at the initial diagnosis was 12.79 ± 3.75 years. Common presenting complaints included heat intolerance (76.6%), excessive sweating (74.5%), palpitations (68.1%), tremors (48.9%), weight loss (38.3%), increased appetite (34%), diarrhea (31.9%), and constipation (4.3%). The mean thyrotropin receptor antibody titer was 16.93 ± 13.47 IU/L. Remission was achieved in two (4.3%) patients treated with antithyroid drugs.

**Conclusion:**

Graves’ disease is the most common cause of juvenile hyperthyroidism, and treating physicians should be aware of its signs and symptoms to avoid treatment delays.

## INTRODUCTION

Hyperthyroidism is less common in children and adolescents when compared with the adult population (^1^). The various causes of hyperthyroidism in the young population include Graves’ disease, Hashimoto’s thyroiditis, pituitary adenomas secreting thyroid-stimulating hormone (TSH), benign toxic adenomas, exogenous hormone consumption, and, rarely, pituitary resistance to thyroid hormones and McCune-Albright syndrome (^2,3^).

Graves’ disease is a rare autoimmune disorder in which thyrotropin receptor antibodies (TRAbs) stimulate the TSH receptor, leading to hyperthyroidism. It accounts for 10 to 15% of all thyroid diseases in childhood (^2^). With an incidence of approximately 0.02% among children and adolescents, Graves’ disease is caused by multiple environmental, genetic, and immune factors (^4^). Dermatologic symptoms and severe ophthalmopathy are not frequently seen in young patients. The treatment options for pediatric Graves’ disease include the use of antithyroid drugs (ATDs) as first-line treatment, and radioactive iodine or total thyroidectomy as definitive treatments.

Considering that the early identification of Graves’ disease in children substantially improves their clinical care, this study was conducted to identify early manifestations of this condition in young patientsand its treatment outcomes.

## SUBJECTS AND METHODS

This hospital-based study analyzed the cases of 62 children and adolescents with Graves’ disease aged between 1 month and 18 years, who visited the Pediatric Endocrinology Department at Indira Gandhi Institute of Child Health, Bengaluru, from 2011 to 2022. The patients’ medical records were reviewed after the institutional Ethics Committee approved the study protocol, and informed consent or assent was obtained from the study participants. All children in the age group defined above, who were diagnosed clinically and biochemically as having Graves’ disease, were included.

Children with thyroid tumors, Hashimoto’s thyroiditis, neonatal Graves’ disease, and drug-induced secondary hyperthyroidism were excluded from the study. Out of the 62 patients identified, 47 met the inclusion criteria and were included in the analysis.

The diagnosis of Graves’ disease was established based on the patients’ clinical presentations, thyroid function tests, and increased uptake of radioactive iodine on thyroid scan. Thyroid function tests indicative of Graves’ disease included elevated serum levels of total T3 (normal values: 80 to 200 ng/dL) and/or free T4 (normal values: 0.8 to 2 ng/dL), along with suppressed TSH levels (normal values: 0.27 to 4.2 µIU/mL) and positive TRAb (normal values: zero to 0.9 IU/L). The thyroid hormone profile was obtained using electrochemiluminescence immunoassay, while an enzyme-linked immunosorbent assay (ELISA) was used for TRAb determination.

Thyroid scan was performed 20 minutes after intravenous injection of Tc-99m pertechnetate (normal values: 0.3 to 3%). Some patients underwent fine-needle aspiration cytology. The participants’ demographics, clinical features, presentation, relevant laboratory records, treatment (type and therapeutic doses of the prescribed ATD and prescription of beta blockers), and outcomes at the end of 2 years of treatment were recorded and analyzed.

Remission was defined as clinical and biochemical euthyroidism for at least 12 months after ATD withdrawal. Relapse was defined as recurrence of symptoms with elevated levels of free T4 and/or free T3 and suppressed TSH requiring ATD and/or leading to definitive therapy within 1 year after stopping the medication (^5,6^). Other parameters considered included the total duration of follow-up, time to achieve euthyroidism, number of relapses, and complications of medical therapy, radioiodine, and surgery.

### Statistical analysis

The collected data were entered into a Microsoft Excel 2016 spreadsheet and analyzed using Statistical Package for the Social Sciences (SPSS) for Windows, version 29.0 (IBM Corp, Armonk, NY, USA). Descriptive statistics, including frequency and percentage analyses, were used for categorical variables, while mean ± standard deviation was used for continuous variables. Data are presented accordingly; p-values < 0.05 were considered significant.

## RESULTS

**Figure 1:**
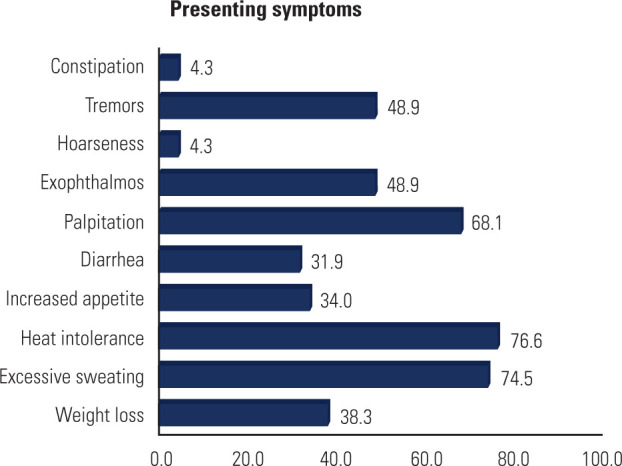
Presenting symptoms among the patients with Graves’ disease included in the present study (shown in percentages).

Among the 47 patients with Graves’ disease included in the study, 31 (66%) were girls, yielding a female-to-male ratio of 1.93:1. Their mean age at initial diagnosis was 12.79 ± 3.75 years (range 3.33 to 16.92 years). Overall, 17 patients (36.2%) presented with symptoms before puberty and 30 (63.8%) during puberty. The most common symptoms at presentation are summarized in **Figure 1**. Goiter was present in 87.3% of the patients; among them, 80.9% had a diffuse goiter and 6.4% had a multinodular goiter. There were 23 cases (48.9%) of exophthalmos, and 30 (63.8%) patients had eye signs. A history of parental consanguinity was present in 11 (23.4%) children, and a family history of hyperthyroidism occurred in 4 (8.5%) cases.

At diagnosis, the mean total T3 was 4.46 ± 2.69 ng/mL, mean free T3 was 15.09 ± 8.96 pg/mL, mean free T4 was 7.04 ± 15.44 ng/dL, and mean TSH was 0.02 µIU/mL. Positive TRAb was detected in 15 (31.9%) patients, and the mean TRAb titer was 16.93 ± 13.47 IU/L. Antithyroid peroxidase (anti-TPO) and antithyroglobulin (anti-TG) antibodies were positive in 27 (57.4%) and 10 (21.3%) cases, respectively.

A baseline complete hemogram and liver function test were obtained from all patients and were normal in all cases. Overall, 33 patients were treated with carbimazole, with a mean dose at treatment initiation of 0.46 ± 0.16 mg/kg/day, and 14 patients were treated with methimazole, with a mean dose at treatment initiation of 0.29 ± 0.14 mg/kg/day. Doses were titrated every 4 to 8 weeks based on the patients’ clinical responses and thyroid function tests. Propranolol was started in 37 (78.7%) patients at a mean dose of 1.06 ± 1.29 mg/kg/day. Seven (14.9%) patients received block-replacement therapy, while the remaining received dose-titration therapy. The mean treatment duration was 2.15 ± 1.53 years, and the mean time to reach euthyroidism was 4.14 ± 1.88 months (range 2 to 8 months).

Arthralgia was reported as a side effect in only one patient, who had been treated with carbimazole, and the ATD dose was subsequently reduced accordingly. After treatment for 2 years, the mean total T3 level was 2.08 ± 1.32 ng/mL, the mean free T4 was 1.78 ± 1.85 ng/dL, and the mean TSH was 2.56 ± 1.59 µIU/mL in the overall sample.

Remission was achieved in two (4.3%) patients on ATD; no relapse occurred in these patients. The remaining patients continued on their medications, as they had not achieved euthyroidism. Subsequently, 13 (27.7%) patients required radioactive iodine ablation, and one patient underwent thyroidectomy. No postoperative surgical complication was observed. All these 14 (29.78%) patients are currently receiving thyroid hormone supplementation.

## DISCUSSION

Graves’ disease is an autoimmune condition with multiple hypermetabolic symptoms due to the interaction of TRAb antibodies with the TSH receptor, leading to increased secretion of thyroid hormones (^7^). In children, Graves’ disease is the most common cause of hyperthyroidism; it is rare before the age of 3 years, and its incidence increases progressively, peaking by adolescence (^8^). The average age at onset of Graves’ disease in our study was 12.79 ± 3.75 years, with the youngest patient being 3.33 years old. Symptom onset has been reported as early as the age of 1.1 years, while the onset of hyperthyroidism is around the age of 9 years of age in most children, according to a study by Mokhashi and cols. (^9,10^). Bhadada and cols. (^11^) reported a female-to-male ratio of 2.1:1, which is comparable with the ratio of 1.93:1 found in our study, with the majority of cases (63.8%) presenting at puberty. Notably, Graves’ disease is more common in peripubertal and pubertal girls. Various studies have suggested a key influence of sex hormones on autoimmune diseases, and among young individuals, girls are known to have a stronger inflammatory response than boys. Estrogen acts via its receptor (Erα) on regulatory T cells and stimulates the immune response, while androgen has an immunoprotective role in this process (^12,13^).

The genetics of hyperthyroidism have been postulated to involve both autosomal recessive and autosomal dominant modes of inheritance. Bhadada and cols. (^11^), Raza and cols. (^14^), and Vaidya and cols. (^15^) have reported a positive family history of hyperthyroidism in 8.9%, 30%, and 37% of their patients, respectively. In the present study, the corresponding rate was 8.5%. It is important for parents to be aware of the signs and symptoms of hyperthyroidism to avoid treatment delays.

Heat intolerance, excessive sweating, palpitation, tremors, weight loss, and increased appetite were frequent manifestations of hyperthyroidism in our patients, which is consistent with reports from other studies (^2,11^). A comparison of the frequency of manifestations at presentation between the present study and similar previous studies is summarized in **Table 1**. Goiter was more frequent in girls, probably because of the increased incidence of hyperthyroidism in them. The incidence of goiter is higher in children and adolescents compared with adults with Graves’ disease, likely due to the stimulatory effect of TSAb and thyroid growth-stimulating immunoglobulins (TGIs) on the thyroid (^16^). Exophthalmos was present in 48.9% of the patients in our study, which is comparable to findings from other studies (^10,11^). Holt and cols. (^17^) noted that children and adolescents have less severe exophthalmos than adults. Behavioral problems like anxiety, mood swings, and sleep disturbance were recorded in some patients in the present study. None of our patients presented with pretibial myxedema (dermopathy), thyroid acropachy, periodic paralysis, or thyroid storm – all of which are rare in children.

**Table 1:** Comparison of the frequencies of Graves’ disease manifestations at presentation among the patients in the present study, compared with those reported in other studies

Manifestations	Present study	LaFranchi & Mandel (^2^)	Bhadada and cols. (^11^)	Raza and cols. (^14^)
Goiter	87.3	99	98.2	98
Weight loss	38.3	54	82.1	54
Tachycardia	68.1	83	80.0	95
Tremor	48.9	61	78.2	51
Eye signs	63.8	66	58.9	71

Results expressed as %.

Zöphel and cols. (^18^) reported that TRAb was positive in approximately 60 to 90% of children with Graves’ disease, which is a higher rate compared with the one found in the present study (31.9%). Notably, the thyroid hormone profile of our patients was comparable to that reported in other studies (^10,11^).

Carbimazole and methimazole were used as first-line ATD in our patients. Studies show a variable range of remissions with ATD, ranging from 33% to 64% (^4,10,11,19^). Remission was achieved only in two (4.3%) patients in our study, probably because of poor compliance among those who did not achieve remission. Predictors of early remission in children with hyperthyroidism are low heart rate, high body mass index, small goiter, low T3 and T4 levels, and low radioiodine uptake (^20,21^). Delayed remission is seen in children when compared with adults due to immunomodulatory effects of puberty and poor compliance with the medications.

Side effects of ATD were seen in only 2.1% of the patients in our study, which is aligned with the finding by Bhadada and cols.(^11^) but much lower than that in other pediatric studies (^4,10^). Block-replacement therapy using methimazole combined with levothyroxine to achieve long-term remission has been attempted in patients with Graves’ disease. Raja and cols. (^14^) have shown that block-replacement therapy was more convenient in juvenile hyperthyroidism than the titration regimen with respect to dose adjustment and yearly hospital visits (p < 0.001).

In Europe, surgery (*i.e.*, subtotal, near-total, or total thyroidectomy) is the therapeutic choice for children and adolescents with Graves’ disease after treatment recurrence during or after ATD, as well as in those who are unable to tolerate ATDs (^2,22^).

Compared with adult patients, pediatric patients have a narrow time gap from diagnosis to treatment with ATDs, and less time from initiation of ATDs to surgery (15 months *versus* 6 months (^23^). Of the 47 patients in our study, only one underwent subtotal thyroidectomy, with no postoperative complications. Radioiodine ablation is used less frequently in Europe than in American centers. In our study, 13 (27.7%) patients received radioiodine ablation, and their rates of hypothyroidism were comparable to those reported in other studies (^14^).

Graves’ disease is the most common cause of juvenile hyperthyroidism, and its manifestations can be subtle and heterogeneous, requiring a strong suspicion level. It is occasionally associated with serious side effects and requires prolonged follow-up. Importantly, increased awareness of the early symptoms of Graves’ disease avoids treatment delays.
